# Key to Xenobiotic Carotenoids

**DOI:** 10.3390/molecules17032877

**Published:** 2012-03-07

**Authors:** Hans-Richard Sliwka, Vassilia Partali

**Affiliations:** Department of Chemistry, Norwegian University of Science and Technology, 7491 Trondheim, Norway

**Keywords:** carotenoids, xanthophylls, fluorocarotenoids, chlorocarotenoids, bromocarotenoids, iodocarotenoids, silicon carotenoids, nitrogen carotenoids, sulfur carotenoids, selenium carotenoids, iron carotenoids

## Abstract

A listing of carotenoids with heteroatoms (X = F, Cl, Br, I, Si, N, S, Se, Fe) directly attached to the carotenoid carbon skeleton has been compiled. The 178 listed carotenoids with C,H,X atoms demonstrate that the classical division of carotenoids into hydrocarbon carotenoids (C,H) and xanthophylls (C,H,O) has become obsolete.

## 1. Introduction

The number of natural occurring carotenoids registered in the relevant books on the topic has increased continuously: 19 carotenoids in 1934, 67 in 1948, 273 in 1971, 563 in 1987, 750 in 2004 [1,2,3,4,5]. The importance of the Carotenoids Handbook is evident for all those working frequently or occasionally with carotenoids. However, the extensive compilation of natural occurring carotenoids has ignored the existence of the numerous xenobiotic carotenoids [6]. The impact of the Carotenoids Handbook is overwhelming insofar that carotenoids with atoms other than C,H,O are barely thinkable. Carotenoids are still classified in two groups: *carotenes* (polyenes containing C,H) and *xanthophylls* (polyenes with C,H,O), and the occurrence of carotenoids with other atoms was not contemplated by the existing nomenclature rules. In contrast, the Natural Product Reports dedicate a specific chapter to steroids with heteroelements, sulfur flavonoids and heteroatom-substituted carbohydrates have been reviewed. [7,8,9,10]. Admittedly, no hetero-carotenoids have been detected so far in Nature, but nonetheless, it is not incongruous to expect carotenoids from sea organisms to incorporate Cl (compounds **5Cl-8Cl** in the list) [11]; the interactions between selenium and carotenoids support speculations about the existence of combination products [12,13,14]. After all, heterocarotenoids may not keep forever their status as xenobiotic compounds, though by then, xenophobia towards xenobiotic carotenoids may be encountered. In a historical review on the “Development of Carotenoid Chemistry 1922–1991” the first Br-, N- and S-carotenoids (**4Br-9Br**, **2N**, **12S**) were ignored [15]. When the author’s first manuscript on carotenoid thioketones (**1S-3S**) was rejected by the referees, the honorary co-author commented the rejection as the logical consequence of working with bizarre compounds. The syntheses of selenium carotenoids (**1Se-7Se**) were regarded by some of the author’s colleagues as a completely useless, ill-famed and ill-smelling occupation. Strangely enough, the summarizing speaker at the end of a carotenoid conference intentionally omitted to mention the author’s presentation on S, N and Se carotenoids. Fortunately, these narrow-minded discriminatory prejudices have now tended to cease; heterocarotenoids have found applications impossible to achieve with “normal” carotenoids, e.g., **2S**, **15S**, **3Se**, **12N**, **46N** [16,17,18,19].

Despite the increasing interest in xenobiotic carotenoids, searching the databases for these compounds often results in zero hits. The unawareness of heterocarotenoids may perhaps be the reason for avoidable syntheses. The molecular wire carotenoid thiol **15S** was prepared in several steps [16]. Carotenoid thione **2S**, synthesized previously from a commercial carotenoid in a one-step synthesis, could probably have been more appropriate for the investigation [20]. Even an author sensitized to xenobiotic carotenoids witnessed ignorance; compounds **25N**, **27N**, **29N** were not cited in a paper on carotenoid oxime hydrochlorides **19N-22N** [21,22]. Unfamiliarity with heterocarotenoids is possibly the cause for further lack of mention, e.g., nitrile carotenoid **6N** was patented in 1990 and published in 2011 without referring to previous work from 1988; thienyl carotenoid **3◉S**, first reported in 1981, was not cited when the compound was resynthesized 20 years later (for an explanation of the designation **3S** see Section 4: Nomenclature).

This thematic issue of *Molecules* on “Carotenoids” now offers the opportunity to compile a systematic listing of xenobiotic carotenoids. This inventory is a first attempt to take these carotenoids out of their obscurity. 

## 2. Historical Remarks

Carotenoids became eye-catching in 1906 with the invention of chromatography by Tswett and got scientific consecration with the first determination of their molecular formula by Willstäter in 1907 [23,24]. During the period of structure determination the first nitrogen carotenoids were prepared as analytical derivatives (oxime, semicarbazone) [25,26]. Bromo and sulfur carotenoids were synthesized in 1958 and 1959 and chloro carotenoids in 1976 [27,28,29]. The synthesis of carotenoid amines was not successful until 1990 [30,31,32]. The most heterogenic carotenoids are probably iron carbonyl compounds **5Fe-7Fe**. The common Greek-letter termed cyclic end groups are now increasingly being replaced by heterocycles.

## 3. Selection Criteria

Polyenes with a branched polyene chain >C20 capped with different cyclic or acyclic end groups and with heteroatoms covalently bound to the carbon carotenoid were considered. Thus, compounds with a heteroatom linked via oxygen to the carotenoid scaffold were omitted (e.g., phosphates). Adhering strictly to the isoprenoid nature of carotenoids would not allow including the interesting aza compound **37N** [33]. This compound has been perceived as an azine of retinal, but is much more attractive when viewed as a diazapolyene. Various carotenoid derivatives prepared for analytical purposes (oximes, hydrazones amides *etc.*) are not mentioned [34,35], unless the derivative has also found an application extending characterization, e.g., canthaxanthin oxime was skipped, canthaxanthin oxime hydrochloride **21N** as a surface active hydrophilic carotenoid was included [22]. Some carotenoids are drawn in the concise all-*trans* form, since the dimension of the actual *cis*-isomers would be too space demanding, e.g., **32N** and **33N**. The main concern of the recording, the heteroatom character of the compound, is not affected by this presentation. 

In a departure from *Molecules’* normal style, reference registration in the compound list follows the example of the handbooks excluding article title and search-irrelevant data on the length of a paper. The references for the individual compounds are not exhaustive. A reader interested in a particular compound should perform a structural search in a database to receive complete and updated citations. 

There certainly exist more xenobiotic carotenoids than presented in the list. Many hetero-carotenoids, especially from the patent literature, are not recorded owing to search problems or involuntary neglect. Such compounds ought to be included in a forthcoming extended register. Enlarging the selection criteria to <C20 chains, to xenobiotic C,H,O carotenoids, considering heteroatoms outside the carotenoid carbon skeleton sphere and taking into account ionic bounded heteroatoms is desirable for future compilation [36,37]. It would furthermore be valuable to have at hand a complete directory of isotope-substituted carotenoids (D, T, ^13^C, ^14^C) [38,39,40]. A catalog of modified carotenoids (e.g., long chain carotenoids, carotenoid dimers, carotenoids with deviated conjugation, hydrophilic carotenoids) and of compounds where carotenoids are part of other molecule classes (e.g., carotenoid lipids, antioxidant combinations) would likewise be desirable [41,42,43,44,45,46,47,48,49,50,51].

## 4. Nomenclature

The designation “xenobiotic carotenoids” is synonymously used with the term “heterocarotenoids”.

Whereas “heterocarotenoids” may appear more precise, the prefix hetero- is too strongly linked with heterocyclic chemistry and could create confusing expressions such as heterocyclic heterocarotenoids. Xenobiotic is, at present, the more explanatory designation. 

Applying the nomenclature rules to xenobiotic carotenoids can lead to unintelligible descriptions; consequently, many authors have avoided naming their products, e.g., **11Cl**-**15Cl** [52]. Keeping in mind that a short trivial name engenders more associative information than a (semi)systematic designation, some names in the list may appear randomly chosen or meaningless. A name search in a database will, therefore, often be unsuccessful, e.g., the name dicyano-C48:15 for **9N** is certainly not canonical, but articulates the essential information: a dicyano substituted carotenoid of 48 C with 15 conjugated double bonds. The exact name would hide this evidence. In any case, the interested reader should certainly scrutinize the carotenoids visually and not by their appellation, and a structure search in a database is, therefore, recommended. The structures are approximately listed according to increasing structural complexity; however, the relation to a parent compound was considered more important than complexity ranking.

Aryl carotenoids have been recorded separately within a heteroatom section. Natural occurring aryl carotenoids display trimethylbenzene ɸ− or χ− end groups. Phenyl end groups without methyl are identified as either 16,17,18-trinor-ɸ− or 16,17,18-trinor-χ−; nevertheless, the letter ɸ is preferred, in analogy to the widely used short form of ɸ for °phenyl [53]. Thus, all carotenoids with a benzene ring are termed ɸ-carotenoids; the ɸ-ring positions are indicated as recommended by the nomenclature rule.

The compounds were arbitrarily numbered; the numbers are not intended to reflect the appointed personal identification digits used in the Key to Carotenoids and the Carotenoids Handbook [4,5]. Carotenoids with heterocycles were, for example, enumerated as x◉S, ◉S indicating a cycle with sulfur.

The catalog of xenobiotic carotenoids definitely proves that the term*xanthophyll* has become obsolete [54]. Applying the classical two level differentiation − hydrocarbon carotenoids (C, H) and xanthophylls (C,H,O)—simply implies denying the existence of the listed 178 carotenoids. The use of *xanthophyll* is therefore discouraged and should be replaced by *oxygen carotenoids*; such a designation is unequivocally extendable to *sulfur (nitrogen, halogen…) carotenoids*.

## 5. Conclusions

Xenobiotic carotenoids have been synthesized for a long time but have remained largely unnoticed by carotenoid chemists. Many of those who work with these compounds may not consider themselves carotenoid chemists. Heteroatoms have helped carotenoids to leave their terrain of origin: xenobiotic carotenoids merit the same appreciation as biotic carotenoids.

## 6. List of Xenobiotic Carotenoids

### 6.1. Halogen-Carotenoids

#### 6.1.1. Fluorine **F**

1F 9-trifluoromethyl-β,β-carotene C_40_H_53_F_3_


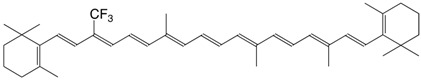


D. Hoischen, L.U. Colmenares, I. Koukhareva, M. Ho, R.S.H. Liu, *J. Fluorine Chem*. **1999**, *97*, 165

2F 13-trifluoromethyl-β,β-carotene C_40_H_53_F_3_


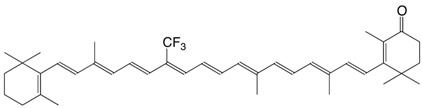


D. Hoischen, L.U. Colmenares, I. Koukhareva, M. Ho, R.S.H. Liu, *J. Fluorine Chem*. **1999**, *97*, 165

3F 9,9'-bis(trifluoromethyl)-β-carotene C_40_H_50_F_6_


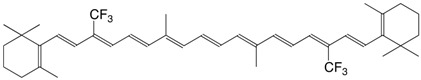


D. Hoischen, L.U. Colmenares, I. Koukhareva, M. Ho, R.S.H. Liu, *J. Fluorine Chem*. **1999**, *97*, 165

4F 13,13'-bis(trifluoromethyl)-β-carotene C_40_H_50_F_6_


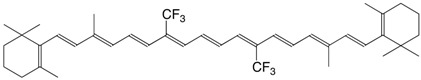


D. Hoischen, L.U. Colmenares, I. Koukhareva, M. Ho, R.S.H. Liu, *J. Fluorine Chem*. **1999**, *97*, 165

5F 9-trifluoromethyl echinenone C_40_H_51_F_3_O


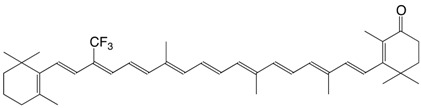


D. Hoischen, L.U. Colmenares, I. Koukhareva, M. Ho, R.S.H. Liu, *J. Fluorine Chem*. **1999**, *97*, 165

6F 13-trifluoromethyl echinenone C_40_H_51_F_3_O 


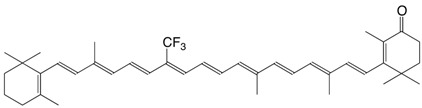


D. Hoischen, L.U. Colmenares, I. Koukhareva, M. Ho, R.S.H. Liu, *J. Fluorine Chem*. **1999**, *97*, 165

7F 9-trifluoromethyl canthaxanthin C_40_H_49_F_3_O_2_


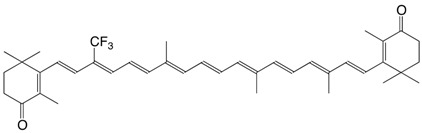


D. Hoischen, L.U. Colmenares, I. Koukhareva, M. Ho, R.S.H. Liu, *J. Fluorine Chem*. **1999**, *97*, 165

8F 13-trifluoromethyl canthaxanthin C_40_H_49_F_3_O_2_


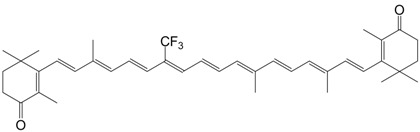


D. Hoischen, L.U. Colmenares, I. Koukhareva, M. Ho, R.S.H. Liu, *J. Fluorine Chem*. **1999**, *97*, 165

9F 3,3'-difluoro-canthaxanthin C_40_H_50_F_2_O_2_


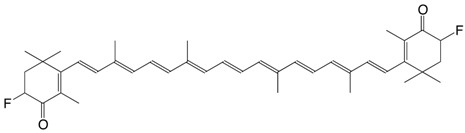


R.S.H. Liu, J. Liu, *J. Nat. Prod.* **2011**, *74*, 512

10F 10-fluoro-astaxanthin C_40_H_51_FO_4_


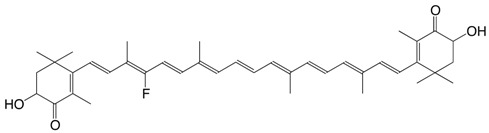


R.S.H. Liu, J. Liu, *J. Nat. Prod.* **2011**, *74*, 512

11F 14-fluoro-astaxanthin C_40_H_51_FO_4_


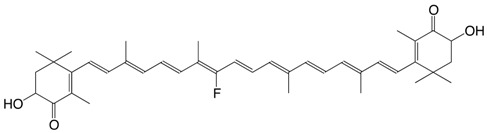


R.S.H. Liu, J. Liu, *J. Nat. Prod.* **2011**, *74*, 512

12F 3,3',10,10',14-pentafluoro-canthaxanthin C_40_H_47_F_5_O_2_


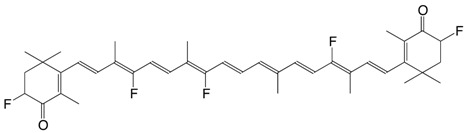


R.S.H. Liu, J. Liu, *J. Nat. Prod.* **2011**, *74*,512

ɸ-carotenoids

13F 1',2',3',4',5'-pentafluoro-β,ɸ-carotene C_37_H_41_F_5_


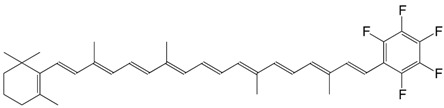


E. Hand, K.A. Belmore, L.D. Kispert, *Helv. Chim. Acta* **1993**, *76*, 1928

#### 6.1.2. Chlorine **Cl**

1Cl crocetin dichloride C_20_H_22_Cl_2_O_2_


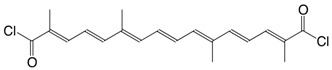


H. Pfander, F. Wittwer, *Helv. Chim. Acta* **1979**, *62*, 1944

2Cl norbixin dichloride C_24_H_26_Cl_2_O_2_


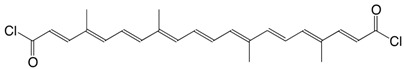


L. Levy, R.H. Binnington, A. Tabatnik, *WO 02/068385*, **2002**

3Cl bixin chloride C_25_H_29_ClO_3_


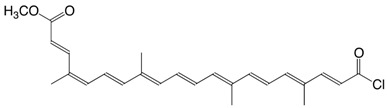


G. Ferrari, V. Vecchietti, *EP 30009*, **1981**

T. Komatsu, E. Tsuchia, C. Böttcher, D. Donner, C. Messerschmidt, U. Siggel, W. Stocker, J.P. Rabe, J.H. Fuhrhop, *J. Am. Chem. Soc*. **1997**, *119*, 11660

4Cl β-apo-8'-carotenoyl chloride, C30-acid chloride C_30_H_39_ClO


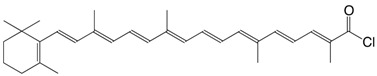


T. Naalsund, K.E. Malterrud, V. Partali, H.R. Sliwka, *Chem. Phys. Lipids* **2001**, *112*, 59

L. Levy, R. H. Binnington, A. Tabatnik, *WO 02/068385*, **2002**

5Cl 4-chloro-β,β-carotene C_40_H_55_Cl


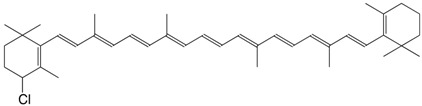


H. Pfander, U. Leuenberger, *Chimia* **1976**, *30*, 71

6Cl 4-chloro-3',4'-didehydro-β,β-carotene C_40_H_53_Cl


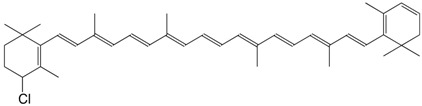


H. Pfander, U. Leuenberger, *Chimia* **1976**, *30*, 71

7Cl 4'-chloro-β,β-caroten-3-ol C_40_H_55_ClO


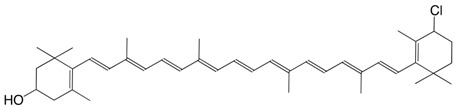


H. Pfander, U. Leuenberger, *Chimia* **1976**, *30*, 71

8Cl 4,4'-dichloro-β,β-carotene C_40_H_54_Cl_2_


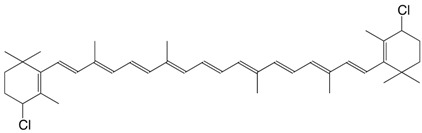


H. Pfander, U. Leuenberger, *Chimia* **1976**, *30*, 71

9Cl 5,5'-dichloro-4,5,4',5'-tetrahydroisocarotene C_41_H_58_Cl_2_


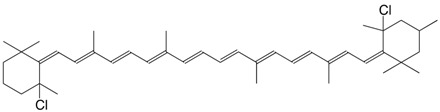


C. Bodea, E. Nicoara, Acad. rep. populare Romîne, Filiala Cluj, Studii Cercetâri Chim. **1959**, 10, 1959

10Cl 7-chloro-mutatoxanthin-3,3'-diacetate C_44_H_59_ClO_5_


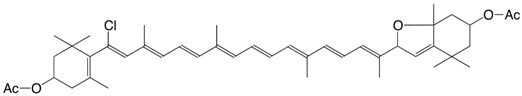


J.E. Johansen, S. Liaaen-Jensen, *Acta Chem. Scand*. **1974**, *B28*, 949

R. Buchecker, S. Liaaen-Jensen, *Helv. Chim. Acta* **1975**, *58*, 89

11Cl 3'-chloro-4,5-dehydro-5-dehydroxy-neochrome C_37_H_49_ClO_2_


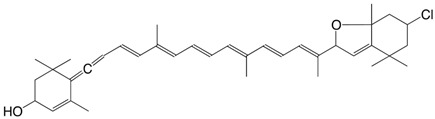


R. Buchecker, S. Liaaen-Jensen, *Helv. Chim.* *Acta* **1975**, *58*, 89

12Cl 3'-chloro-2,3-didehydro-5,18-dehydro-5-dehydroxy-neochrome C_37_H_47_ClO


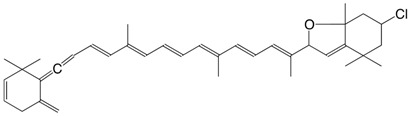


R. Buchecker, S. Liaaen-Jensen, *Helv. Chim. Acta* **1975**, *58*, 89

13Cl 7-chloro-mutatoxanthin-19',11'-olide 3-acetate C_42_H_53_ClO_6_


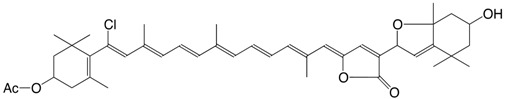


J.E. Johansen, S. Liaaen-Jensen, *Acta Chem. Scand*. **1974**, *B28*, 949

14Cl 3'-chloro-6,7-didehydro-peridinol-3-acetate C_42_H_53_ClO_6_


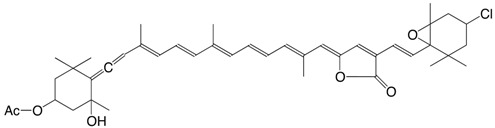


J.E. Johansen, S. Liaaen-Jensen, *Acta Chem. Scand*. **1974**, *B28*, 949

15Cl 4,5-didehydro-5-dehydroxy-3'-chloro-peridinin-3-acetate C_42_H_51_ClO_5_


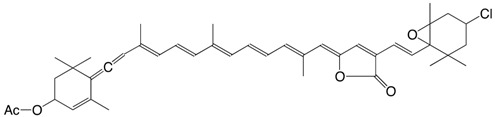


J.E. Johansen, S. Liaaen-Jensen, *Acta Chem. Scand*. **1974**, *B28*, 949

#### 6.1.3. Bromine **Br**

1Br 20-bromo-crocetindial C_20_H_23_BrO_2_


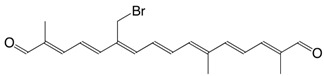


J.E. Johansen, S. Liaaen-Jensen, *Acta Chem. Scand.* **1975**, *B29*, 315

2Br 20,20'-dibromo-crocetindial C_20_H_22_Br_2_O_2_


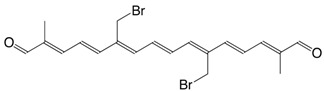


J.E. Johansen, S. Liaaen-Jensen, *Acta Chem. Scand.* **1975**, *B29*, 315

3Br 4-bromo-β,β-carotene C_40_H_55_Br


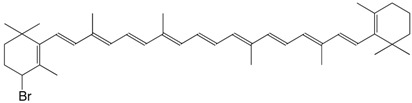


R. Entschel, P. Karrer, *Helv. Chim. Acta* **1958**, *41*, 983

J. Morel, *DE2001957*, **1970**

4 Br 4,4'-dibromo-β,β-carotene C_40_H_58_Br_2_


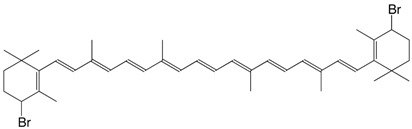


R. Entschel, P. Karrer, *Helv. Chim. Acta* **1958**, *41*, 402

C. Martin, P. Karrer, *Helv. Chim. Acta* **1959**, *42*, 464

5Br 4-bromo-4'-ethoxy-β,β-carotene C_42_H_59_BrO 


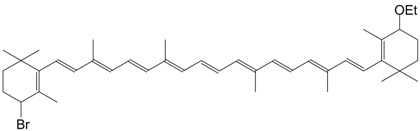


R. Entschel, P. Karrer, *Helv. Chim. Acta* **1958**, *41*, 402 

6Br 4-bromo-4'-ethoxy-β,β-carotene C_44_H_63_BrO_2_


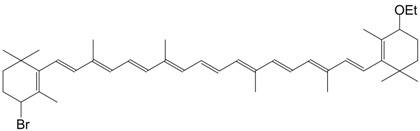


R. Entschel, P. Karrer, *Helv. Chim. Acta* **1958**, *41*, 402

7Br 4-bromo-4-ethoxy echinenone C_42_H_57_BrO_2_


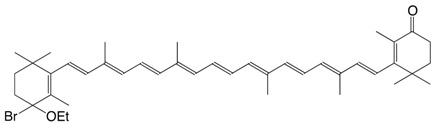


R. Entschel, P. Karrer, *Helv. Chim. Acta* **1958**, *41*, 402

8Br 4 bromo-4',4'-diethoxy-β,β-carotene C_44_H_63_BrO_2_


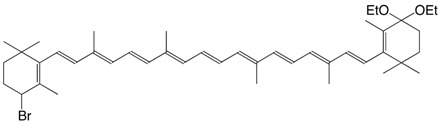


F.J. Petracek, L. Zechmeister, *J. Am.* *Chem. Soc*. **1956**, *78*, 1427

9Br 4,4,4'-tribromo-β,β-carotene C_40_H_53_Br_3_


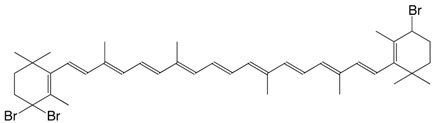


F.J. Petracek, L. Zechmeister, *J. Am. Chem. Soc*. **1956**, *78*, 1427

10Br 7-bromo-mutatoxanthin-diacetate C_44_H_59_BrO_5_


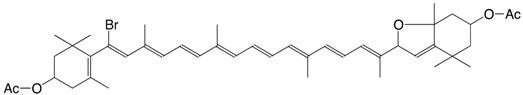


R. Buchecker, S. Liaaen-Jensen, *Helv. Chim.* *Acta* **1975**, *58*, 89

#### 6.1.4. Iodine **I**

1I 5,5'-diiodo-5,6,5',6'-didehydro-β,β-carotene C_40_H_58_I_2_


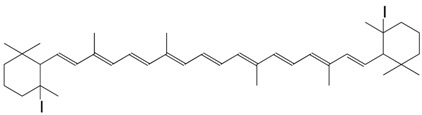


unconfirmed structure

B.G. Savinov, G.S. Tretyakova, *Vitaminy Akad.* *Nauk Ukr. S.S.R* **1953**, *1*, 137

Other carotenoid-iodine compounds are formulated as ionic complexes:

B.F. Lutnaes, J. Krane, S. Liaaen-Jensen, *Org.* *Biomol. Chem*. **2004**, *2*, 2821

### 6.2. Silicon-Carotenoids **Si**

1Si (= 2◉S) 7,5'-diapo-7-thienyl-carotene-5'-triethoxysilane C_34_H_46_O_3_SSi


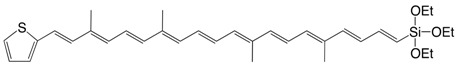


F. Effenberger, M. Wezstein, *Synthesis* **2001**, 1368

### 6.3. Nitrogen-Carotenoids **N**

1N 8'-apo-β-carotene-8'-nitrile C_30_H_39_N


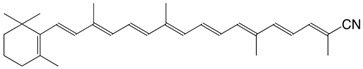


Z. He, D. Gosztola, Y. Deng, G. Gao, M.R. Wasielewski, L.D. Kispert, *J. Phys. Chem. B* **2000**, *104*, 6668

2N 10'-apo-5,6-seco-β-carotene-10'-nitrile C_27_H_35_NO_2_


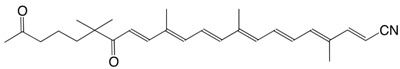


R. Kuhn, H. Brockmann, *Chem. Ber*. **1934**, *67*, 885

3N 6'-apo-β-carotene-6'-nitrile C_32_H_41_N


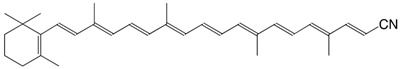


Z. He, D. Gosztola, Y. Deng, G. Gao, M.R. Wasielewski, L.D. Kispert, *J. Phys.* *Chem.* *B* **2000**, *104*, 6668

S. Tretiak, V. Chernyak, S. Mukamel, *J. Am. Chem. Soc*. **1997**, *119*, 11408

S. Gilmour, S.R. Marder, B.G. Tiemann, L.T. Cheng, *J. Chem. Soc. Chem. Commun*. **1993**, 432

4N 4'-apo-β-carotene-4'-nitrile C_34_H_43_N


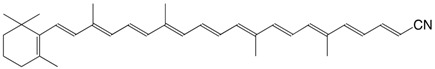


Z. He, D. Gosztola, Y. Deng, G. Gao, M.R. Wasielewski, L.D. Kispert, *J. Phys. Chem.B***2000**, *104*, 6668

5N 7'-cyano-β-apo-7'-carotenoic acid methyl ester C_34_H_43_NO_2_


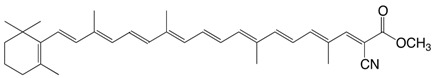


H. Ikeda, T. Sakai, Y. Kawabe, *JP 2-2534*, **1990**

6N 7'-apo-7',7'-dicyano-β−carotene C_33_H_40_N_2_


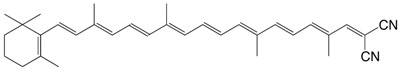


M. Blanchard-Desce, I. Ledoux, J.M. Lehn, J. Malthête, J. Zyss, *J. Chem. Soc. Chem. Commun*. **1988**, 737

H. Ikeda, T. Sakai, Y. Kawabe, *JP 2-2534*, **1990**

M.P. O’Neil, M.R. Nasielewski, M.M. Khaled, L.D. Kispert, *J. Chem.Phys. B*, **1991**, *95*, 7212 

S. Gilmour, S.R. Marder, B.G. Tiemann, L.T. Cheng, *J. Chem. Soc. Chem. Commun*. **1993**, 432

E.S. Hand, K.A. Belmore, L.D. Kispert, *J. Chem.Soc. Perkin Trans 2*, **1993**, 659

S. Tretiak, V. Chernyak, S. Mukamel, *J. Am. Chem. Soc*. **1997**, *119*, 11408

A.J. Cruz, K. Siam, D.P. Rillema, *J. Phys. Chem*. **2011**, *115*, 1108

7N 4,14'-dicyano-20,20’-dinor-β,β-carotene- C_40_H_50_N_2_


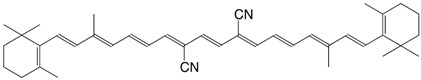


H.H. Haeck, T. Kralt, *Rec. Trav. Chim. Pays-Bas* **1966**, *85*, 343

P.B. Braun, J. Hornstra, J.I. Leenhouts, *Acta Cryst*. **1971**, *B27*, 90

8N dicyano-C44:14 C_48_H_60_N_2_


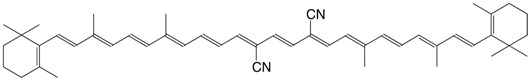


H.H. Haeck, T. Kralt, *Rec. Trav. Chim. Pays-Bas* **1966**, *85*, 343

9N dicyano-C48:15 C_52_H_66_N_2_


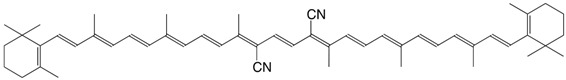


H.H. Haeck, T. Kralt, *Rec. Trav. Chim. Pays-Bas* **1966**, *85*, 343

10N (3*S*)-2',3’-didehydro-β,β-carotene-3-amine C_40_H_55_N


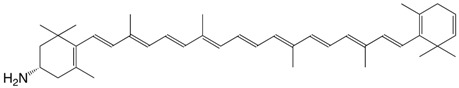


H.R. Sliwka, S. Liaaen-Jensen, *Tetrahedron Asym*. **1993**, *4*, 2377

11N (3*R,*3*'S*)-3'-amino-β,β-carotene-3-ol C_40_H_57_NO


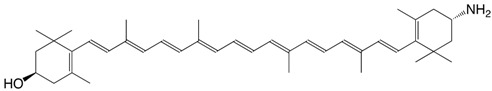


H.R. Sliwka, S. Liaaen-Jensen, *Tetrahedron Asym*. **1993**, *4*, 2377

12N (3*S,*3*'S*)-β,β-carotene-3,3'-amine C_40_H_58_N_2_


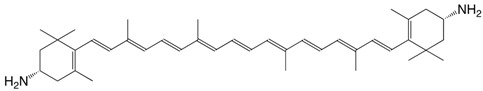


H.R. Sliwka, S. Liaaen-Jensen, *Tetrahedron Asym*. **1993**, *4*, 2377

J. Inananga, M. Yamaguchi, *Mem. Fac. Sci. Kyushi Univ. Ser. C*, **1989**, *17*, 109

13N (3*S,*3*'S*)-β,β-carotene-3,3'-diacetamide C_44_H_62_N_2_O_2_


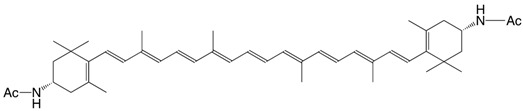


J. Inananga, M. Yamaguchi, *Mem. Fac. Sci.* *Kyushi Univ. Ser. C*, **1989**, *17*, 109

14N 4,4'-dianilino-β,β-carotene C_52_H_66_N_2_


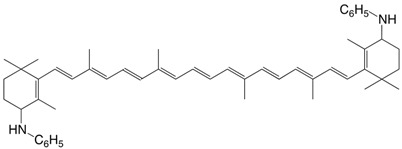


C. Martin, P. Karrer, *Helv. Chim. Acta* **1959**, *42*, 464

H. Budzikiewicz, H.Brzezinka, B. Johannes, *Monatshefte* **1970**, *101*, 579

15N 4,4'-bis(*N*-methyl-anilino)-β,β-carotene C_54_H_70_N_2_


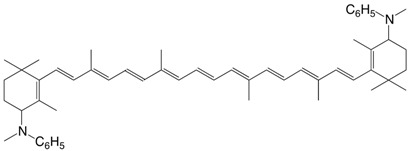


C. Martin, P. Karrer, *Helv. Chim. Acta* **1959**, *42*, 464

16N (3*S*)-3-azido-2',3'-didehydro-β,β-carotene C_40_H_53_N_3_


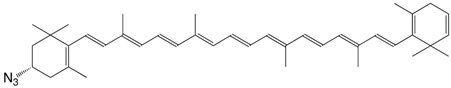


H.R. Sliwka, S. Liaaen-Jensen, *Tetrahedron Asym*. **1993**, *4*, 2377

H.R. Sliwka, *Helv. Chim. Acta* **1999**, *82*, 161

17N (3*R*,3'*S*)-3'-azido-β,β-carotene-3-ol C_40_H_55_N_3_O


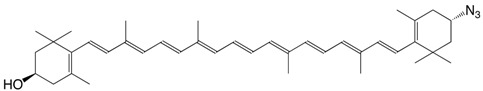


H.R. Sliwka, S. Liaaen-Jensen, *Tetrahedron Asym*. **1993**, *4*, 2377

H.R. Sliwka, *Helv. Chim. Acta* **1999**, *82*, 161

18N (3*S,*3*'S*)-diazido-β,β-carotene C_40_H_54_N_6_


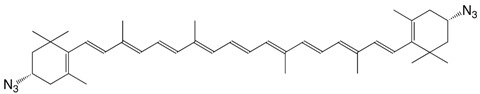


H.R. Sliwka, S. Liaaen-Jensen, *Tetrahedron Asym*. **1993**, *4*, 2377

J. Inananga, M. Yamaguchi, *Mem. Fac. Sci. Kyushi Univ. Ser. C*, **1989**, *17*, 109

H.R. Sliwka, *Helv. Chim. Acta* **1999**, *82*, 161

19N 8'-apo-β-caroten-8'-aldoxime hydrochloride, C30-aldoxime hydrochloride C_30_H_42_ClNO


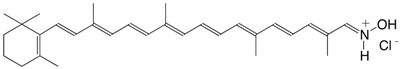


J. Willibald, S. Rennebaum, S. Breukers, S.H. Abdel Hafez, A. Patel, C.L. Øpstad, R. Schmid, S. Nalum Naess, H.R. Sliwka, V. Partali, *Chem. Phys. Lipids* **2009**, *161*, 32

H.R. Sliwka, V. Partali, S.F. Lockwood, in *Carotenoids*, ed. J.T. Landrum, CRC Press, Boca Raton, USA, **2010**, chpt. 3

20N echinenenon oxime hydrochloride C_40_H_56_ClNO


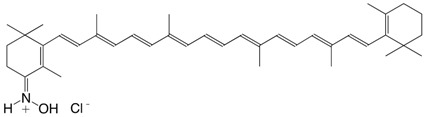


J. Willibald, S. Rennebaum, S. Breukers, S.H. Abdel Hafez, A. Patel, C.L. Øpstad, R. Schmid, S. Nalum Naess, H.R. Sliwka, V. Partali, *Chem. Phys. Lipids* **2009**, *161*, 32

H.R. Sliwka, V. Partali, S.F. Lockwood, in *Carotenoids*, ed. J.T. Landrum, CRC Press, Boca Raton, USA, **2010**, chpt. 3

21N canthaxanthin dioxime hydrochloride C_40_H_56_Cl_2_N_2_O_2_


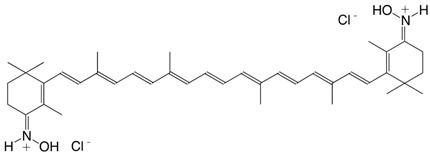


J. Willibald, S. Rennebaum, S. Breukers, S.H. Abdel Hafez, A. Patel, C.L. Øpstad, R. Schmid, S. Nalum Naess, H.R. Sliwka, V. Partali, *Chem. Phys. Lipids* **2009**, *161*, 32

H.R. Sliwka, V. Partali, S.F. Lockwood, in *Carotenoids*, ed. J.T. Landrum, CRC Press, Boca Raton, USA, **2010**, chpt. 3

22N astaxanthin dioxime hydrochloride C_40_H_56_Cl_2_N_2_O_4_


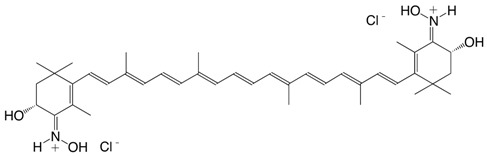


J. Willibald, S. Rennebaum, S. Breukers, S.H. Abdel Hafez, A. Patel, C.L. Øpstad, R. Schmid, S. Nalum Naess, H.R. Sliwka, V. Partali, *Chem. Phys. Lipids* **2009**, *161*, 32

H.R. Sliwka, V. Partali, S.F. Lockwood, in *Carotenoids*, ed. J.T. Landrum, CRC Press, Boca Raton, USA, **2010**, chpt. 3

23N 7'-aza-7'-methyl-7'-apo-β-carotene C_31_H_43_N


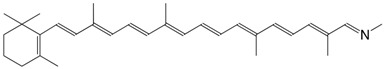


G.A.J. Pitt, F.D. Collins, R.A. Morton, P. Stok, *Biochem. J.* **1955**, *59*, 122

24N 7'-aza-7'-butyl-7'-apo-β-carotene C_34_H_49_N


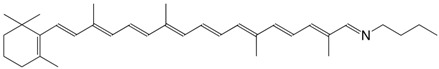


T.A. Moore, P.S. Song, *J. Mol. Spec*. **1974**, *52*, 224

25N 7'-aza-7'-butyl-7'-apo-β-carotene hydrochloride C_35_H_52_ClN


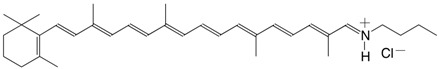


T.A. Moore, P.S. Song, *J. Mol. Spec*. **1974**, *52*, 224

26N 5'-aza-5'-butyl-5'-apo-β-carotene C_37_H_53_N


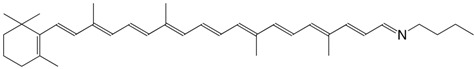


T.A. Moore, P.S. Song, *J. Mol. Spec*. **1974**, *52*, 224

27N 5'-aza-5'-butyl-5'-apo-β-carotene hydrochloride C_37_H_54_ClN


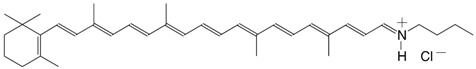


T.A. Moore, P.S. Song, *J. Mol. Spec*. **1974**, *52*, 224

28N 5'-aza-5'-butyl-5'-apo-β-caroten-3-ol C_36_H_51_NO


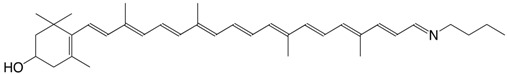


T.A. Moore, P.S. Song, *J. Mol. Spec*. **1974**, *52*, 224

29N 5'-aza-5'-butyl-5'-apo-β-caroten-3-ol hydrochloride C_36_H_52_ClNO


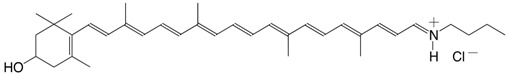


T.A. Moore, P.S. Song, *J. Mol. Spec*. **1974**, *52*, 224

30N 7'-aza-7'-ureido-7'-apo-β-caroten-3-ol, β-citraurin semicarbazone C_31_H_43_N_3_O_2_


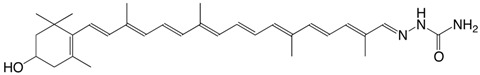


L. Zechmeister, L. von Cholnoky, *Liebigs* *Ann*. **1937**, *530*, 291

31N 7'-nitro-7'-apo-β-carotene C_31_H_41_NO_2_


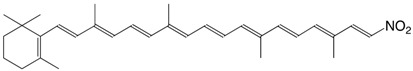


H. Ikeda, T. Sakai, Y. Kawabe, *JP 2-2534*, **1990**

32N 4-nitro-β,β-carotene C_40_H_55_NO_2_


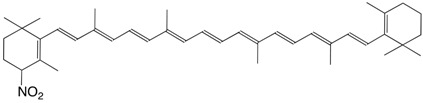


D.L. Baker, E.S. Kroll, N. Jacobsen, D.C. Liebler, *Chem. Res. Toxicol.* **1999**, *12*, 535

33N 11-nitroastaxanthin C_40_H_51_NO_6_


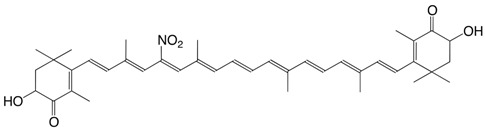


R. Yoshioka, T. Hayakawa, K. Ishuzuka, A. Kulkarni, Y. Terada, T. Maoka, H. Etoh, *Tetrahedron Lett*. **2006**, *47*, 3637 (*cis*-isomer)

34N 15-nitroastaxanthin C_40_H_51_NO_6_


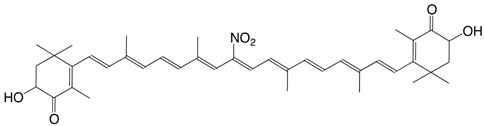


R. Yoshioka, T. Hayakawa, K. Ishuzuka, A. Kulkarni, Y. Terada, T. Maoka, H. Etoh, *Tetrahedron Lett*. **2006**, *47*, 3637 (*cis*-isomer)

35N 12-nitrocapsanthin C_40_H_55_NO_5_


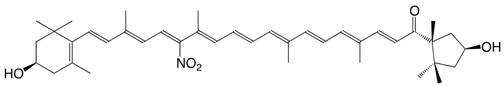


M. Tsuboi, H. Etoh, K. Kato, H. Nakatugawa, H. Kato, Y. Maejima, G. Matsumoto, H. Mori, M. Hosokawa, K. Miyashita, H. Tokuda, N. Suzui, T. Maoka, *J. Agric. Food Chem*. **2011**, *59*, 10572

36N 11-nitrofucoxanthin C_42_H_57_NO_8_


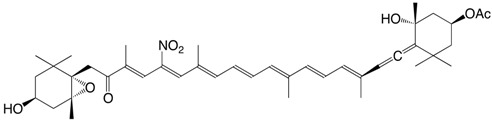


M. Tsuboi, H. Etoh, K. Kato, H. Nakatugawa, H. Kato, Y. Maejima, G. Matsumoto, H. Mori, M. Hosokawa, K. Miyashita, H. Tokuda, N. Suzui, T. Maoka, *J. Agric. Food Chem*. **2011**, *59*, 10572

37N 15-nitrofucoxanthin C_42_H_57_NO_8_


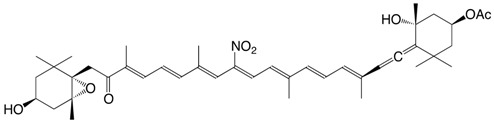


M. Tsuboi, H. Etoh, K. Kato, H. Nakatugawa, H. Kato, Y. Maejima, G. Matsumoto, H. Mori, M. Hosokawa, K. Miyashita, H. Tokuda, N. Suzui, T. Maoka, *J. Agric. Food Chem*. **2011**, *59*, 10572

38N 16,16'-diaza-β,β-carotene (diretinyliden hydrazine) C_40_H_56_N_2_


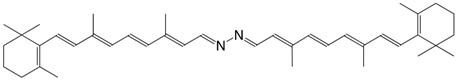


T. Miki, Y. Hara, *JP 34-002118*, **1959**

T. Miki, Y. Hara, *Chem. Pharm. Bull*. **1962**, *10*, 922

39N 5-acetyl-2-nor-β-apo-7'-carotenoic acid amide C_32_H_41_NO_2_


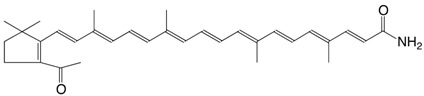


R. Kuhn, H. Brockmann, *Chem. Ber*. **1934**, *67*, 885

40N *N*-hexyl crocetinamide C_26_H_35_NO_3_


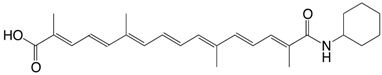


G. Quinkert, K.R. Schmieder, G. Dürner, K. Hache, A. Stegk, D.H.R. Barton, *Chem. Ber*. **1977**, *110*, 3582

41N β-apo-7'-benzoylamino-7'-carotenoic acid *N,N*-diethylamide C_43_H_56_N_2_O_2_


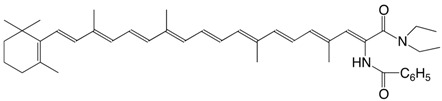


M. Tomoaia-Cotisel, J. Zsako, E. Chifu, D.A. Ladenhead, *Langmuir* **1990,***6*,191. The authors list several related amides.

42N β-apo-7'-benzoylamido-7'-carotenoic acid *N*-aminoethylamide C_41_H_53_N_3_O_2_


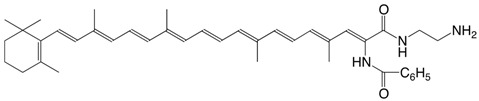


M. Tomoaia-Cotisel, J. Zsako, E. Chifu, D.A. Ladenhead, *Langmuir* **1990,***6*,191

43N β-apo-7'-benzoylamido-7'-carotenoic acid *N*-methyl-N-(2-hydroxyethyl)-amide C_42_H_54_N_2_O_3_


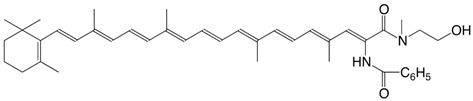


M. Tomoaia-Cotisel, J. Zsako, E. Chifu, D.A. Ladenhead, *Langmuir* **1990,***6*,191

44N β-apo-7'-benzoylamido-7'-carotenoic acid *N,N*-(bis(2-hydroxyethyl)-amide C_43_H_56_N_2_O_4_


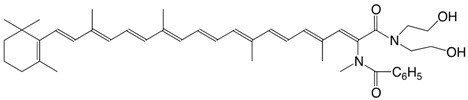


M. Tomoaia-Cotisel, J. Zsako, E. Chifu, D.A. Ladenhead, *Langmuir* **1990,***6*,191

45N *N*-octadecyl bixinamide C_42_H_65_NO_3_


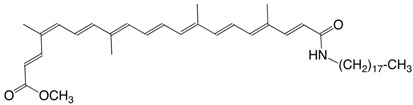


J.H. Fuhrhop, M. Krull, A. Schulz, D. Möbius, *Langmuir* **1990**, *6*,497. The authors list several related bixin amides.

G. Ferrari, V. Vecchietti, *EP 030009*, **1980 **describe numerous bixin amides.

46N dibixine diphenylenediamid C_54_H_60_N_2_O_6_


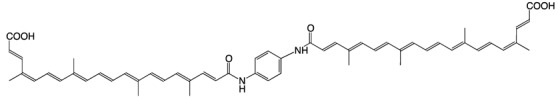


J.H. Fuhrhop, M. Krull, A. Schulz, D. Möbius, *Langmuir* **1990**, *6*, 497

47N imine of tris-(8,8'-diapo-ψ,ψ-carotene-8.8'-diimine) C_72_H_96_N_8_


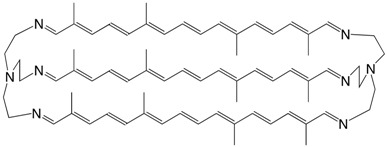


J.M. Lehn, J.P. Vigneron, I. Bkouhe-Waksman, J. Guilhem, C. Pascal, *Helv. Chim. Acta* **1992**, *75*, 1069

ɸ-carotenoids 

48N 7'-aza-3'-methyl-β,ɸ-carotene C_37_H_47_N


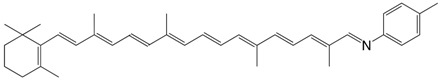


H. Kamogawa, *Polym. Lett. Ed.* **1972**, *10*, 929

49N 7'-aza-3'-dimethylamino-β,ɸ-carotene C_36_H_46_N_2_


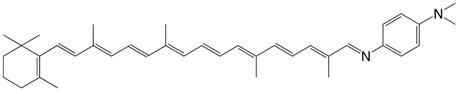


H. Ikeda, T. Sakai, Y. Kawabe, *JP 2-2534*, **1990**

50N 7'-aza-β,ɸ-caroten-3'-amine C_36_H_46_N_2_


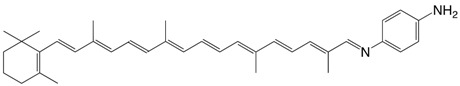


H. Ikeda, T. Sakai, Y. Kawabe, *JP 2-2534*, **1990**

51N 7'-aza-3'-methoxy-β,ɸ-carotene C_37_H_47_NO


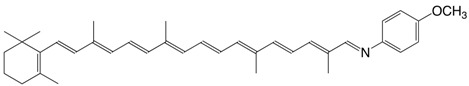


H. Ikeda, T. Sakai, Y. Kawabe, *JP 2-2534*, **1990**

52N β,ɸ-carotene-3'-acetamide C_39_H_49_NO


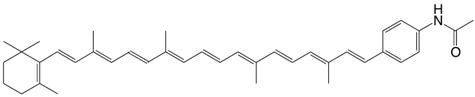


M.R. Wasielewski, P.A. Liddel, D. Barrett, T.A. Moore, D. Gust, *Nature* **1986**, *322*, 570

53N β,ɸ-carotene-3'-porphyrinamide 


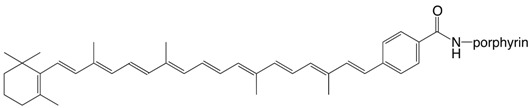


D. Gust, T.A. Moore, P.A. Liddell, G.A. Nemeth, L.R. Makings, A.L. Moore, D. Barrett, P.J. Pessiki, R.V. Bemasson, M. Rougiée, C. Chachaty, F.C. De Schryver, M. Van der Auweraer, A.R. Holzwarth, J. S. Connolly, *J. Am. Chem. Soc*. **1987**, *109*, 846

54N 3'-nitro-β,ɸ-carotene C_37_H_45_NO_2_


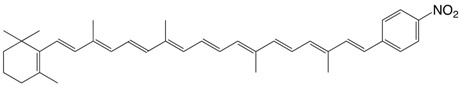


H. Ikeda, T. Sakai, Y. Kawabe, *JP 2-2534*, **1990**

S. Gilmour, S.R. Marder, B.G. Tiemann, L.T. Cheng, *J. Chem. Soc. Chem. Commun*. **1993**, 432

E.S. Hand, K.A. Belmore, L.D. Kispert, *Helv. Chim. Acta* **1993**, *76*, 1928

55N 1',3'-dinitro-β,ɸ-carotene C_37_H_44_N_2_O_4_


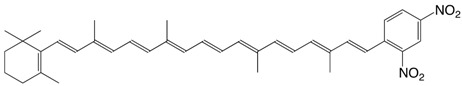


H. Ikeda, T. Sakai, Y. Kawabe, *JP 2-2534*, **1990**

56N 2-dimethylamino-ɸ,ɸ-carotene-3'-nitrile C_37_H_40_N_2_


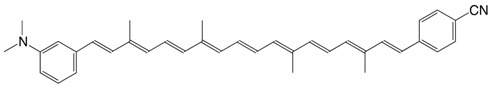


A. Slama-Schwok, M. Blanchard-Desce, J.M Lehn, *J. Phys. Chem*. **1990**, *94*, 3894

57N 3'-nitro-ɸ,ɸ-carotene-2-dimethylamine C_36_H_40_N_2_O_2_


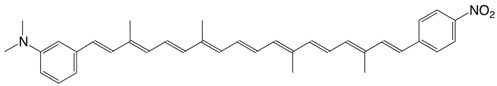


A. Slama-Schwok, M. Blanchard-Desce, J.M Lehn, *J. Phys. Chem*. **1990**, *94*, 3894

58N 7'-cyano-3'-nitro-β,ɸ-carotene C_38_H_44_N_2_O_2_


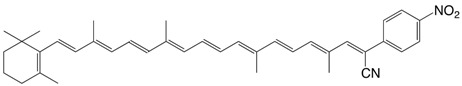


H. Ikeda, T. Sakai, Y. Kawabe, *JP 2-2534*, **1990**

59N 7'-cyano-7'-benzoxo-β-carotene C_39_H_45_NO


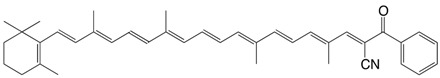


H. Ikeda, T. Sakai, Y. Kawabe, *JP 2-2534*, **1990**

60N 3'-dimethylamino-β,ɸ-carotene C_39_H_51_N


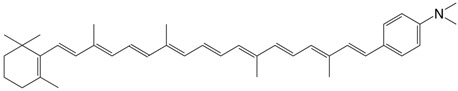


H. Ikeda, T. Sakai, Y. Kawabe, *JP 2-2534*, **1990**

61N 3'-dioctylamino-β,ɸ-carotene C_53_H_79_N


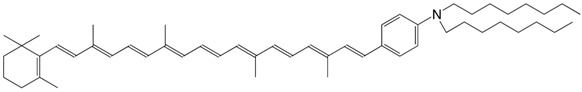


T. Wagner, S. Roth, *Synth. Metals* **1993**, *54*, 307

62N 2',3'-dicyano-β,ɸ-carotene C_39_H_44_N_2_


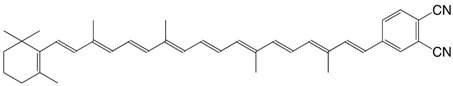


H. Ikeda, T. Sakai, Y. Kawabe, *JP 2-2534*, **1990**

### 6.3. Chalcogen-Carotenoids

#### 6.3.1. Sulfur **S**

1S echinenone thione C_40_H_54_S


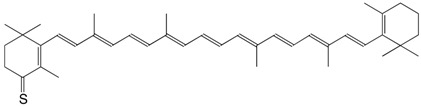


H.R. Sliwka, S. Liaaen-Jensen, *Acta Chem. Scand*. **1994**, *48*, 679

2S canthaxanthin thione C_40_H_52_OS


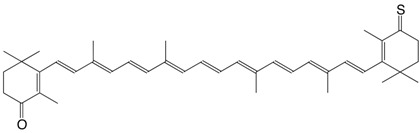


H.R. Sliwka, S. Liaaen-Jensen, *Acta Chem. Scand*. **1994**, *48*, 679

3S rhodoxanthin thione C_40_H_50_OS


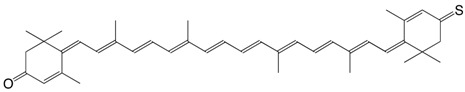


H.R. Sliwka, S. Liaaen-Jensen, *Acta Chem. Scand*. **1994**, *48*, 679

4S 3'-thiolutein C_40_H_56_OS


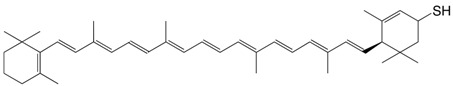


H.R. Sliwka, S. Liaaen-Jensen, *Acta Chem. Scand*. **1990**, *44*, 61 

5S (3*S*)-2',3'-didehydro-β,β-carotene-3-thiol C_40_H_54_S


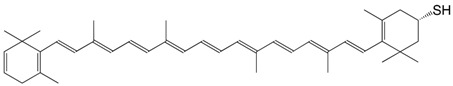


H.R. Sliwka, S. Liaaen-Jensen, *Tetrahdron Asym. ***1993**, *4*, 361 

H.R. Sliwka, *Helv. Chim. Acta* **1999**, *82*, 161

6S (3*R,*3*'S*)-3'-sulfanyl-β,β-caroten-3-ol C_40_H_56_OS


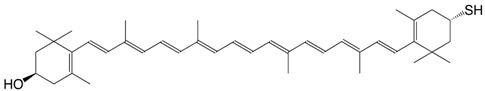


H.R. Sliwka, S. Liaaen-Jensen, *Tetrahdron Asym.* **1993**, *4*, 361 

H.R. Sliwka, *Helv. Chim. Acta* **1999**, *82*, 161

7S 3'-thioacetyl lutein C_42_H_58_O_2_S


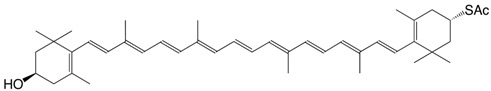


H.R. Sliwka, S. Liaaen-Jensen, *Acta Chem. Scand*. **1990**, *44*, 61 

8S 4,4'-dithioacetyl-β,β-carotene C_44_H_60_O_2_S_2_


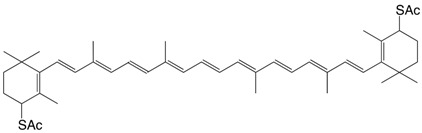


H.R. Sliwka, *Helv. Chim. Acta* **1999**, *82*, 161

9S 4'-thioacethyl-β,β-caroten-4-one C_42_H_56_O_2_S


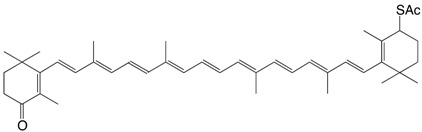


H.R. Sliwka, S. Liaaen-Jensen, *Acta Chem. Scand*. **1990**, *44*, 61

10S (3*R*)-3',4'-didehydro-3-phenylsulfanyl-β,β-carotene C_46_H_58_S


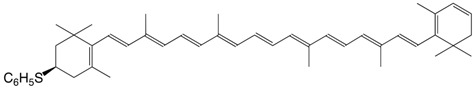


J. Inananga, M. Yamaguchi, *Mem. Fac. Sci. Kyushi Univ. Ser. C*, **1989**, *17*, 109

11S 3,3'-diphenylsulfanyl-β,β-carotene C_52_H_64_S_2_


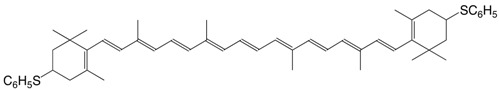


J. Inananga, M. Yamaguchi, *Mem. Fac. Sci. Kyushi Univ. Ser. C*, **1989**, *17*, 109

12S 4,4'-diphenylsulfanyl-β,β-carotene C_52_H_64_S_2_


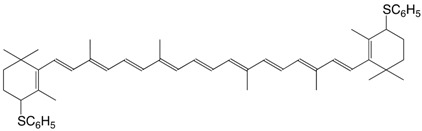


C. Martin, P. Karrer, *Helv. Chim. Acta* **1959**, *42*, 464

13S 3,4'-dehydro-β,β-carotene-4-thioglucopyranoside C_74_H_80_O_9_S


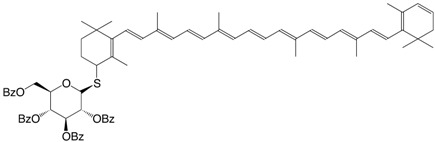


V. Nagy, A. Agócs, E. Turcsi, J. Deli, Tetrahedron Lett. **2010**, *52*, 1020

14S β,β,-carotene-4,4'-bisthioglucopyranoside C_107_H_106_O_18_S_2_


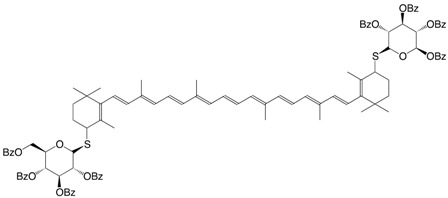


V. Nagy, A. Agócs, E. Turcsi, J. Deli, Tetrahedron Lett. **2010**, *52*, 1020

15S β,β-carotene-15-yl-phenylsulfone C_46_H_60_O_2_S


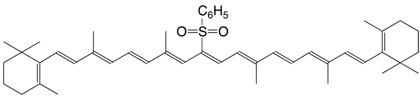


K. Berhard, S. Jäggli, P. Kreienbühl, U. Schwieter, *EU298404*, **1989**. The patent lists numerous other phenylsulfones and *p*-chlorophenylsulfones as synthetic intermediates. 

ɸ-carotenoids 

16S 3-methylsulfanyl-8'-apo-ɸ-carotenal C_28_H_32_OS


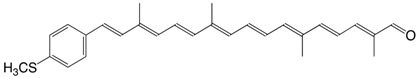


Y.Q. Shen, W. Göhring, S. Hagen, S. Roth, *J. Mol. Electron*. **1990**, *6*, 31

17S β,ɸ-carotene-3'-methanethiol C_38_H_48_S


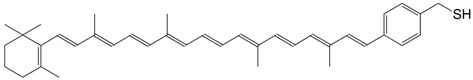


G. Leatherman, E.N. Duranti, D. Gust, T.A. Moore, A.L. Moore, S. Stone, Z. Zhou, P. Rez, Y.Z. Liu, S.M. Lindsay, *J. Phys. Chem. B* **1999**, *103*, 4006

18S 10,10'-dimethyl-13-phenyl-9,9',13,13'-tetranor-ɸ,ɸ−carotene-3,3'-dimethanethiol C_40_H_40_S_2_


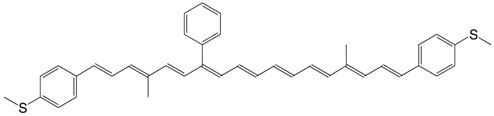


J. Maeng, S.B. Kim, N.J. Lee, E. Choi, S.Y. Jung, I. Hong, S.H. Bae, J.T. Oh, B. Lim, J.W. Kim, C.J. Kang, S. Koo, *Chem. Eur. J*. **2010**, *16*, 7395. The authors list other similar compounds. 

#### 6.3.1. Selenium **Se**

1Se (*3S*)-2',3'-didehydro-β,β-carotene-3-phenylselenide C_46_H_58_Se


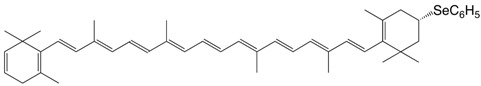


H.R. Sliwka, S. Liaaen-Jensen, *Acta Chem. Scand*. 1**995**, *49*, 428

E. Oliveros, A.M. Braun, T. Aminian-Saghafi, H.R. Sliwka, *New J. Chem*. **1994**, *18*, 535

H.R. Sliwka, *Helv. Chim. Acta* **1999**, *82*, 161

2Se zeaxanthin phenylselenide, (3*R,*3'*S*)-3'-phenylseleno-β,β-caroten-3-ol C_46_H_60_OSe


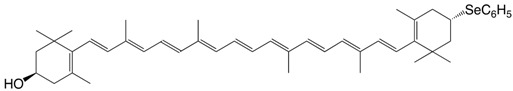


H.R. Sliwka, S. Liaaen-Jensen, *Acta Chem. Scand*. 1**995**, *49*, 428

H.R. Sliwka, *Helv. Chim. Acta* **1999**, *82*, 161

3Se zeaxanthin diphenylselenide, (3*S,*3*'S*)-β,β-carotene-3,3'-diphenylselenide C_52_H_64_Se_2_


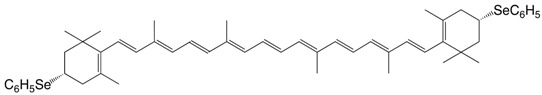


H.R. Sliwka, S. Liaaen-Jensen, *Acta Chem. Scand*. 1**995**, *49*, 428

H.R. Sliwka, *Helv. Chim. Acta* **1999**, *82*, 161

4Se lutein phenylselenide, (3*R,*3*'R,S,*6*'R*)-3'-phenyleleno-β,ε-caroten-3-ol C_46_H_60_OSe


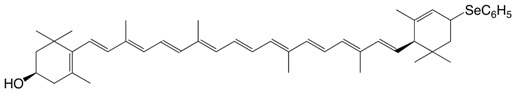


H.R. Sliwka, S. Liaaen-Jensen, *Acta Chem. Scand*. 1**995**, *49*, 428

5Se rhodoxanthin diphenylselenide, 7,7'-di(phenylseleno)-7,8,7',8'-dihydro-*retro*-ε,ε-carotene-

3,3'-dione C_52_H_62_O_2_Se_2_


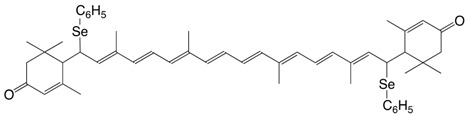


postulated unstable intermediate

H.R. Sliwka, S. Liaaen-Jensen, *Acta Chem. Scand*. 1**995**, *49*, 856

6Se (3*S*)-2',3'-didehydro-β,β-caroten-3-yl-di-O,O-propylselenophosphate C_46_H_67_O_3_PSe


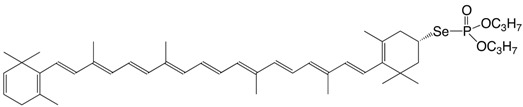


H.R. Sliwka, *Acta Chem. Scand*. 1997, 51, 345

H.R. Sliwka, *Helv. Chim. Acta* **1999**, *82*, 161

7Se (3*R*,3'*S*)-3-hydroxy-3-yl-di-O,O-propylselenophosphate C_46_H_69_O_4_PSe


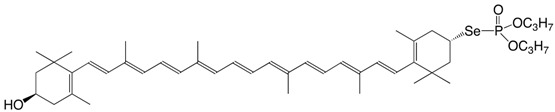


H.R. Sliwka, *Acta Chem. Scand*. 1997, 51, 345

H.R. Sliwka, *Helv. Chim. Acta* **1999**, *82*, 161

### 6.4. Combinations

#### 6.4.1. Nitrogen, Sulfur **N,S**

1NS zeaxanthin thiocyanate, (3*S*)-3'-thiocyano-β,β-caroten-3-ol C_41_H_55_NOS


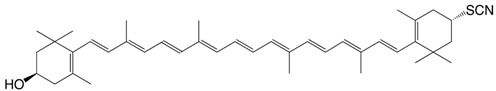


H.R. Sliwka, S. Liaaen-Jensen, *Tetrahedron Asym*. **1993**, *4*, 2377

2NS zeaxanthin dithiocyanat, (3*S*,3'*S*)-β,β-carotene-3,3'-dithiocyanate C_42_H_54_N_2_S_2_


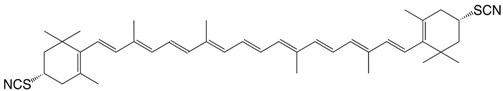


H.R. Sliwka, S. Liaaen-Jensen, *Tetrahedron Asym*. **1993**, *4*, 2377

### 6.5. Iron **Fe**

1Fe ferrocenyl-C21 aldehyde, 7-ferrocenyl-7,8'-diapocaroten-8'-al C_31_H_35_FeO


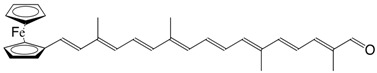


F. Effenberger, H. Schlosser, *Synthesis* **1990**, 1085

2Fe ferrocenyl-C31 aldehyde C_41_H_47_FeO


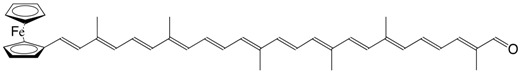


F. Effenberger, H. Schlosser, *Synthesis* **1990**, 1085

3Fe C22 bis(ferrocenyl), 7,7'-bis(ferrocenyl)-7,7'-diapocarotene C_42_H_48_Fe


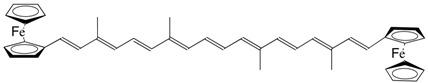


F. Effenberger, H. Schlosser, *Synthesis* **1990**, 1085

4Fe C26 bis(ferrocenyl) C_46_H_52_Fe_2_


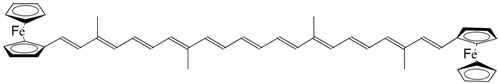


J.M. Lehn, *Angew. Chem. Int. Ed.* **1990**, *29*, 1304 

5Fe tetrakis(iron tricarbonyl)-β,β-carotene C_52_H_56_Fe_4_O_12_


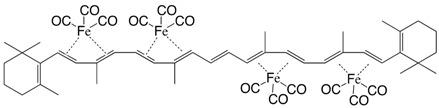


M. Ichikawa, M. Tsutsui, F. Vohwinkel, *Z. Naturforschg*. **1967**, *22b*, 376

6Fe tetrakis(iron tricarbonyl)-β,β-carotene C_52_H_56_Fe_4_O_12_


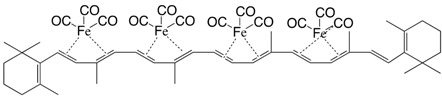


M. Ichikawa, M. Tsutsui, F. Vohwinkel, *Z. Naturforschg*. **1967**, *22b*, 376

7Fe tetrakis(iron tricarbonyl)-β,β-carotene C_52_H_56_Fe_4_O_12_


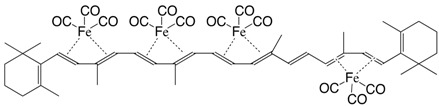


M. Ichikawa, M. Tsutsui, F. Vohwinkel, *Z. Naturforschg*. **1967**, *22b*, 376

### 6.6. Heterocycle Carotenoids

#### 6.6.1. N-heterocycle **◉N**

1◉N crocetin-di-imidazolide C_26_H_30_N_4_O_2_


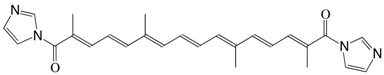


H. Pfander, F. Wittwer, *Helv. Chim. Acta* **1979**, *62*, 1944

2◉N crocetin-bis(1,2,4-triazolide) C_26_H_30_N_4_O_2_


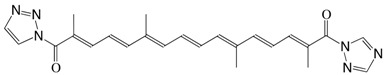


H. Pfander, F. Wittwer, *Helv. Chim. Acta* **1979**, *62*, 1944

3◉N octadexylbixin imidazolide C_45_H_67_N_3_O_2_


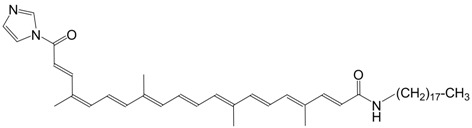


J.H. Fuhrhop, M. Krull, A. Schulz, D. Möbius, *Langmuir* **1990**, *6*,497

4◉N *N*-(8'-apo-β-carotene-8-ylidene)-pyrrolidium perchlorate C_34_H_48_ClNO_4_


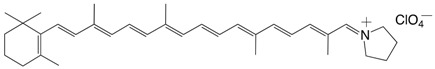


D.L. Coffen, E. Ho, C. Nocka, G. Sasso, V. Toome, T.R. Wagler, T.H. Williams, *J. Prakt. Chem.* **1993**, *335*, 135

5◉N 7'-apo-7'-(*N*-methyl-4-pyridinium)-β-carotene iodide C_37_H_48_IN


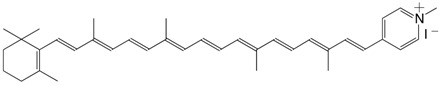


H. Ikeda, T. Sakai, Y. Kawabe, *JP 2-2534*, **1990**

6◉N 7'-apo-7'-(*N*-methyl-2-pyridinium)-β-carotene iodide C_37_H_48_IN


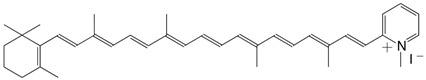


H. Ikeda, T. Sakai, Y. Kawabe, *JP 2-2534*, **1990**

7◉N 7,7'-diapo-7,7'-bis(2-pyridyl)-carotene C_32_H_34_N_2_


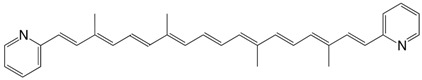


H.R. Brahmana, K. Katsuyama, J. Inaga, T. Katsuki, M. Yamaguchi, *Tetrahedron Lett.* **1981**, *22*, 1695

8◉N 7,7'-diapo-7,7'-bis(3-pyridyl)-carotene C_32_H_34_N_2_


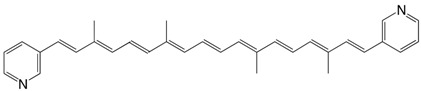


H.R. Brahmana, K. Katsuyama, J. Inaga, T. Katsuki, M. Yamaguchi, *Tetrahedron Lett.* **1981**, *22*, 1695

T.S. Arrhenius, M. Blanchard-Desce, M. Dvolaitzky, J.M. Lehn, J. Malthête, *Proc. Natl. Acad. Sci. USA* **1986**, *83*, 5355

9◉N 7,7'-diapo-7,7'-bis(4-pyridyl)-carotene C_32_H_34_N_2_


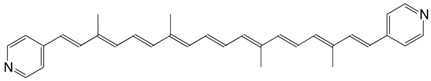


I. Visoly-Fisher, K. Daie, Y. Terazono, C. Herrero, F. Fungo, L. Otero, E. Durantini, J.J. Silber, L. Sereno, D. Gust, T.A. Moore, A.L. Morre, S.M. Lindsay, *PNAS* **2006**, *103*, 8686

10◉N bis(4-pyridyl)-C26:11-carotene C_36_H_38_N_2_


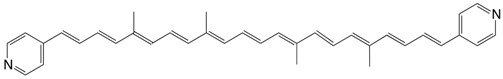


I. Visoly-Fisher, K. Daie, Y. Terazono, C. Herrero, F. Fungo, L. Otero, E. Durantini, J.J. Silber, L. Sereno, D. Gust, T.A. Moore, A.L. Morre, S.M. Lindsay, *PNAS* **2006**, *103*, 8686

11◉N bis(4-pyridyl)-C26:11-carotene C_36_H_38_N_2_


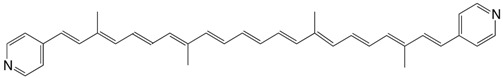


T.S. Arrhenius, M. Blanchard-Desce, M. Dvolaitzky, J.M. Lehn, J. Malthête, *Proc. Natl. Acad. Sci. USA* **1986**, *83*, 5355

12◉N bis(4-pyridyl)-C34:15-carotene C_36_H_38_N_2_


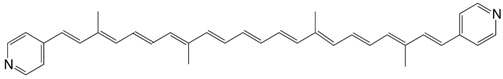


T.S. Arrhenius, M. Blanchard-Desce, M. Dvolaitzky, J.M. Lehn, J. Malthête, *Proc. Natl. Acad. Sci. USA* **1986**, *83*, 5355

13◉N 1,4-bis(4-pyridyl-C12:5)-benzene C_40_H_40_N_2_


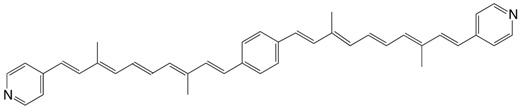


T.S. Arrhenius, M. Blanchard-Desce, M. Dvolaitzky, J.M. Lehn, J. Malthête, *Proc. Natl. Acad. Sci. USA* **1986**, *83*, 5355

14◉N 1,4-bis(4-pyridinium-C12:5)-benzene diiodide C_42_H_46_I_2_N_2_


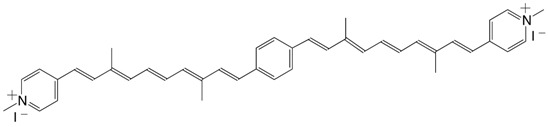


T.S. Arrhenius, M. Blanchard-Desce, M. Dvolaitzky, J.M. Lehn, J. Malthête, *Proc. Natl. Acad. Sci. USA* **1986**, *83*, 5355

15◉N 7,7'-diapo-7,7'-bis(4-methylpyridinium)-carotene diiodide C_34_H_40_I_2_N_2_


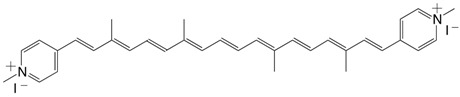


T.S. Arrhenius, M. Blanchard-Desce, M. Dvolaitzky, J.M. Lehn, J. Malthête, *Proc. Natl. Acad. Sci. USA* **1986**, *83*, 5355

16◉N 7,7'-diapo-7,7'-bis(4-ethylpyridinium)-carotene dibromide C_36_H_44_Br_2_N_2_


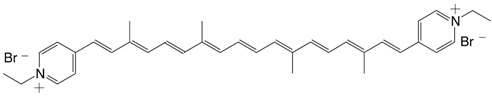


T. Okumoto, N. Morita, I. Nakamura, M. Konishi, M. Yamaguchi, *J. Cancer Res.. Clin. Oncol*. **1985**, *109*, 257

17◉N bis(4-pyridinium)-C34:15-carotene diiodide C_38_H_44_I_2_N_2_


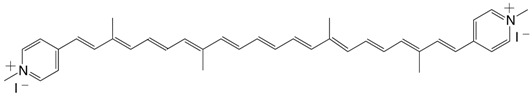


T.S. Arrhenius, M. Blanchard-Desce, M. Dvolaitzky, J.M. Lehn, J. Malthête, *Proc. Natl. Acad. Sci. USA* **1986**, *83*, 5355

18◉N 7,7'-diapo-7,7'-bis(1,3,3 trimethylindolenium)-carotene dichlorate C_42_H_48_Cl_2_N_2_O_8_


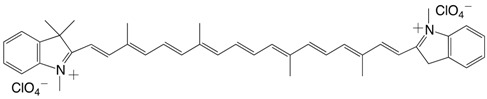


S. Hünig, F. Linhart, D. Scheutzow, *Liebigs Ann.* **1975**, 2089

19◉N 7,7'-diapo-7'-(4-pyridyl)-ɸ-carotene-2-dimethylamine C_35_H_40_N_2_


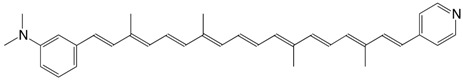


M. Blanchard-Desce, J.M. Lehn, I. Ledoux, J. Zyss, *Special Publication - Royal Society of Chemistry* (Org. Mater. Non-linear Opt.) **1989**, *69*, 170

20◉N 7'-cyano-7'-(4-pyridyl)-β,ɸ-carotene C_37_H_44_N_2_


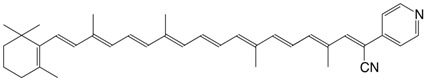


A.J. Cruz, K. Siam, D.P. Rillema, *J. Phys. Chem*. **2011**, *115*, 1108

21◉N lutein-6*H*-1,2-oxazine C_39_H_53_NO_3_


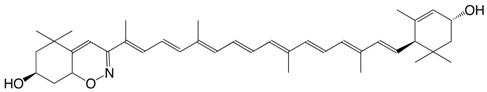


M. Tsuboi, H. Etoh, Y. Yomoda, K. Kato, H. Kato, A. Kulkarni, Y. terada, T. Maoka, H. Mori, T. Inakuma, *Tetrahedron Lett*. **2010**, *521*, 676

22◉N 6,6'-diapocarotenal-6'-(2-phenyl-azlactone) C_33_H_33_NO_3_


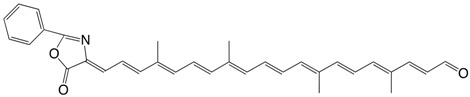


J. Zsako, M. Tomoaia-Cotisel, V. Tamas, C. Coman, E. Chifu, *Rev. Roum. Chim*. **1987**, *32*, 1193. 

The authors describe several other azlactones.

23◉N β-apo-6'-carotenal-2-phenyl-azlactone C_39_H_45_NO_2_


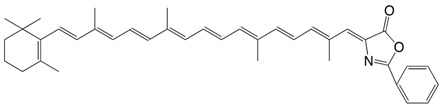


V. Tamas, V. Ciurdaru, C. Bodea, *Rev. Roum. Chim*. **1973**, *18*, 1409

24◉N β-apo-6'-carotendial bis-2-phenyl diazlactone, C24:11 diazlactone C_42_H_38_N_2_O_4_


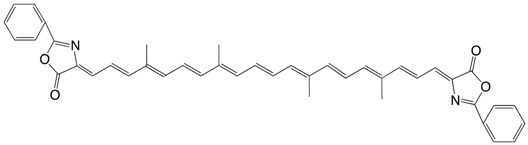


V. Tamas, V. Ciurdaru, C. Bodea, *Rev. Roum. Chim*. **1973**, *18*, 1409

25◉N C30:13-diazlactone C_48_H_48_N_2_O_4_


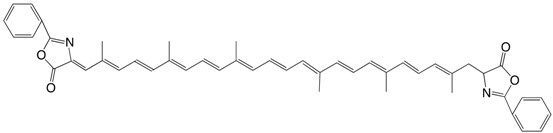


V. Tamas, V. Ciurdaru, C. Bodea, *Rev. Roum. Chim*. **1973**, *18*, 1409. The authors describe several other azlactones.

26◉N bis(2,2'-bipyridine-4-yl)-carotene C_42_H_40_N_4_


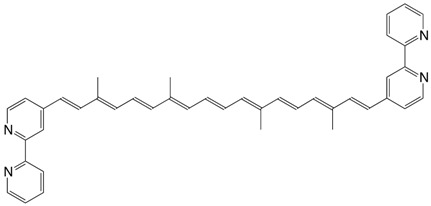


F. Effenberger, M. Wezstein, *Synthesis* **2001**, 1368

27◉N astacene bisphenazine C_52_H_56_N_4_


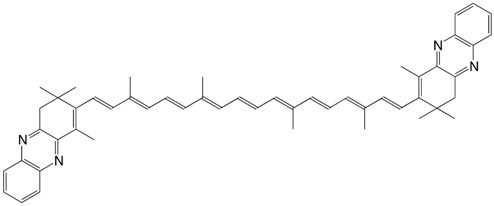


P. Karrer, L. Loewe, *Helv. Chim. Acta* **1934**, *17*, 745

S. Hertzberg, S. Liaaen-Jensen, C.R. Enzell, G.W. Francis, *Acta Chem. Scand*. **1969**, *23*, 3290

28◉N violerythrin bisquinoxaline C_50_H_52_N_4_


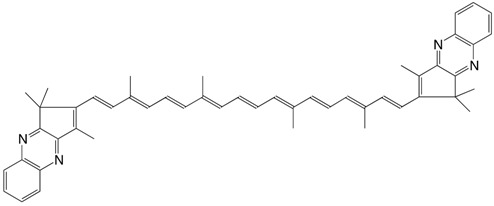


S. Hertzberg, S. Liaaen-Jensen, C.R. Enzell, G.W. Francis, *Acta Chem. Scand*. **1969**, *23*, 3290

29◉N 7,7'-diapo-7',7'-dicyano-7-julolidinyl-carotene C_36_H_39_N_3_


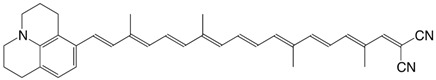


M. Blanchard-Desce, J.M. Lehn, M. Barzoukas, I. Ledoux, J. Zyss, *Chem. Phys*. **1994**, *181*, 281

30◉N 7'-apo-3-cyano-7'-julolidinyl-ɸ−carotene C_41_H_44_N_2_


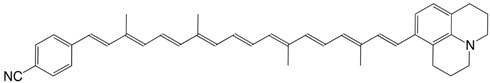


M. Blanchard-Desce, J.M. Lehn, M. Barzoukas, I. Ledoux, J. Zyss, *Chem. Phys*. **1994**, 181, 281

31◉N 7'-apo-7'-julolidinyl-3-nitro-ɸ-carotene C_40_H_44_N_2_O_2_


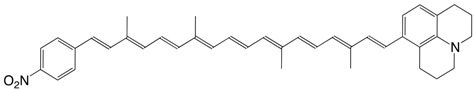


M. Blanchard-Desce, J.M. Lehn, M. Barzoukas, I. Ledoux, J. Zyss, *Chem. Phys*. **1994**, 181, 281

32◉N diterpyridine carotenoid C_52_H_46_N_6_


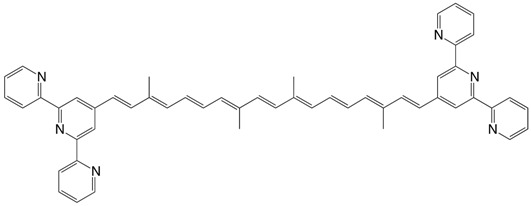


G. Pickaert, R. Ziessel, *Tetrahedron Lett*. **1998**, *39*, 3497

#### 6.6.2. S-heterocycle **◉S**

1◉S 3,4,3',4',-tetrahydrospirilloxanthin-20-(1,3-dithiolane) C_44_H_66_O_2_S_2_


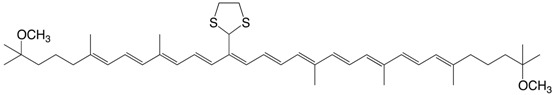


A.J. Aasen, S. Liaaen Jensen, *Acta Chem. Scand*. **1967**, *21*, 2185

2◉S (= 1Si) 7,5'-diapo-7-thienyl-carotene-5'-triethoxysilane C_34_H_46_O_3_SSi


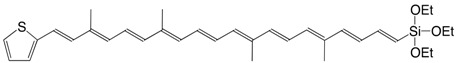


F. Effenberger, M. Wezstein, *Synthesis* **2001**, 1368

3◉S 7,7'-diapo-bis(2-thienyl)-carotene C_30_H_32_S_2_


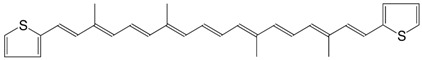


H.R. Brahmana, K. Katsuyama, J. Inaga, T. Katsuki, M. Yamaguchi, *Tetrahedron Lett*. **1981**, *22*, 1695

F. Effenberger, M. Wezstein, *Synthesis* **2001**, 1368

4◉S 7,7'-diapo-bis(3-thienyl)-carotene C_30_H_32_S_2_


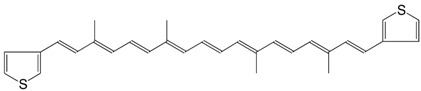


H.R. Brahmana, K. Katsuyama, J. Inaga, T. Katsuki, M. Yamaguchi, *Tetrahedron Lett*. **1981**, *22*, 1695

5◉S 7',8-diapo-7',7'-dicyano-8-(benzodithiol-2-ylidene)-carotene C_30_H_28_N_2_S_2_


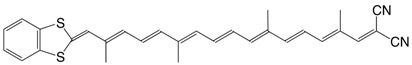


M. Blanchard-Desce, I. Ledoux, J. Malthête, J. Zyss, *J. Chem. Soc., Chem. Commun*. **1988**, 737

6◉S 8'-apo-8'-(benzodithiol-2-ylidene)-3-cyano-ɸ−carotene C_35_H_33_NS_2_


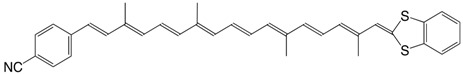


M. Blanchard-Desce, I. Ledoux, J. Malthête, J. Zyss, *J. Chem. Soc., Chem. Commun*. **1988**, 737

7◉S 8'-apo-8'-(benzodithiol-2-ylidene)-3-nitro-ɸ-carotene C_34_H_33_NO_2_S_2_


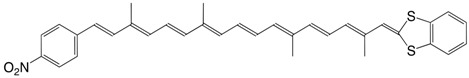


M. Blanchard-Desce, I. Ledoux, J. Malthête, J. Zyss, *J. Chem. Soc., Chem. Commun*. **1988**, 737

#### 6.6.3. N,S-heterocycle **◉N,S**

1◉N,S 7,8'-diapo-8'-(benzodithiol-2-ylidene)-7-(4-pyridyl)-carotene C_33_H_33_NS_2_


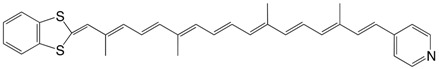


M. Blanchard-Desce, I. Ledoux, J. Malthête, J. Zyss, *J. Chem. Soc., Chem. Commun*. **1988**, 737

2◉N,S 7,8'-diapo-8'-(benzodithiol-2-ylidene)-7-(4-pyridinium)-carotene iodide C_34_H_36_INS_2_


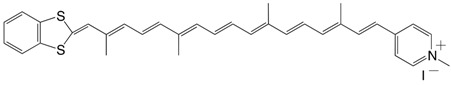


M. Blanchard-Desce, I. Ledoux, J. Malthête, J. Zyss, *J. Chem. Soc., Chem. Commun*. **1988**, 737

3◉N,S C30-aldehyde rhodanine, 8'-apo-β-carotenyliden-rhodanine C_33_H_41_NOS_2_


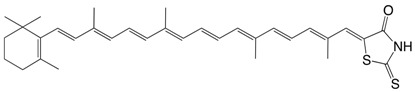


H. Hegedus, *US3071583*, **1963**

H. Thommen, *Int. Z. Vitaminforsch*. **1967**, *37*, 175
